# Distribution of large lungworms (Nematoda: Dictyocaulidae) in free-roaming populations of red deer *Cervus elaphus* (L.) with the description of *Dictyocaulus skrjabini* n. sp.

**DOI:** 10.1017/S003118202300080X

**Published:** 2023-09

**Authors:** Anna Maria Pyziel, Zdzisław Laskowski, Daniel Klich, Aleksander Wiaczesław Demiaszkiewicz, Stanisław Kaczor, Dorota Merta, Janusz Kobielski, Julita Nowakowska, Krzysztof Anusz, Johan Höglund

**Affiliations:** 1 Department of Food Hygiene and Public Health Protection, Institute of Veterinary Medicine, Warsaw University of Life Sciences (WULS-SGGW), Warsaw, Poland; 2 Polish Academy of Sciences, W. Stefański Institute of Parasitology, Warsaw, Poland; 3 Department of Animal Genetics and Conservation, Institute of Animal Sciences, Warsaw University of Life Sciences (WULS-SGGW), Warsaw, Poland; 4 County Veterinary Inspectorate in Sanok, Sanok, Poland; 5 Institute of Biology and Earth Sciences, Pedagogical University of Cracow, Kraków, Poland; 6 Forest Inspectorate in Pieńsk, Pieńsk, Poland; 7 Institute of Biology, University of Warsaw, Imaging Laboratory, Warsaw, Poland; 8 Department of Biomedical Sciences and Veterinary Public Health (BVF), Division of Parasitology, Swedish University of Agricultural Sciences (SLU), Uppsala, Sweden

**Keywords:** *Dictyocaulus skrjabini* n. sp, fallow deer (*Dama dama*), moose (*Alces alces*), phylogenetic reconstruction, red deer (*Cervus elaphus*)

## Abstract

Lungworms of the genus *Dictyocaulus* are causative agents of parasitic bronchitis in domestic and wild ungulates. This study investigates the distribution, morphology and genetic diversity of *D. cervi* and a new lungworm species, *Dictyocaulus skrjabini* n. sp. infecting red deer *Cervus elaphus*, fallow deer *Dama dama* and moose *Alces alces* in Poland and Sweden. The study was conducted on 167 red deer from Poland and on the DNA of lungworms derived from 7 fallow deer, 4 red deer and 2 moose collected in Sweden. The prevalence of *D. cervi* and *D. skrjabini* n. sp. in dissected red deer in Poland was 31.1% and 7.2%, respectively. Moreover, *D. skrjabini* n. sp. was confirmed molecularly in 7 isolates of fallow deer lungworms and 1 isolate of red deer lungworms from Sweden. *Dictyocaulus skrjabini* n. sp. was established based on combination of their distinct molecular and morphological features; these included the length of cephalic vesicle, buccal capsule (BC), buccal capsule wall (BCW), distance from anterior extremity to the nerve ring, the width of head, oesophagus, cephalic vesicle, BC and BCW, as well as the dimensions of reproductive organs of male and female. Additionally, molecular analyses revealed 0.9% nucleotide sequence divergence for 1,605 bp *SSU* rDNA, and 16.5–17.3% nucleotide sequence divergence for 642 bp mitochondrial *cyt*B between *D. skrjabini* n. sp. and *D. cervi*, respectively, and 18.7–19% between *D. skrjabini* n. sp. and *D. eckerti*, which translates into 18.2–18.7% amino acid sequence divergence between *D. skrjabini* n. sp. and both lungworms.

## Graphical abstract

**Figure:**
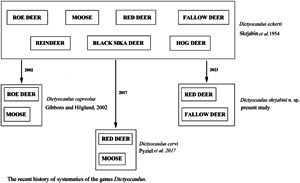

## Introduction

Lungworms of the genus *Dictyocaulus* Railliet and Henry, 1907 (Nematoda: Trichostrongyloidea) are causative agents of parasitic bronchitis in domestic and wild ungulates (Hӧglund *et al*., 2003; Ács *et al*., 2016; Pyziel *et al*., 2018*a*). In the first systematic revision of the genus, *D. eckerti* Skrjabin, 1931 was described from a reindeer (Skrjabin *et al*., 1954). The most current systematic revision of the genus maintained *D. eckerti* as a collective species for all cervids (Gibbons and Khalil, 1988). Following the retrospective description of *D. capreolus* Gibbons and Höglund, 2002 from roe deer and moose, and *D. cervi* Pyziel, Laskowski, Demiaszkiewicz and Höglund, 2017 from red deer, the classification was revised, and the species were separated from *D. eckerti* (Gibbons and Hӧglund, 2002; Pyziel *et al*., 2017).

*Dictyocaulus cervi* was described on the basis of the unique ribosomal *SSU*, *ITS*2 and mitochondrial *cox*1 sequences, as well as on the morphological characteristics of male and female lungworms obtained from red deer inhabiting north-eastern Poland. Interestingly, *D. cervi* was also detected in wild red deer in 2 areas in the Italian Alps (Cafiso *et al*., 2023), as well as in a rocky mountain elk *Cervus canadensis nelsoni* in the USA (Bangoura *et al*., 2021). Moreover, some nucleotide sequences derived from lungworms of red deer from Hungary were found to match sequences of *D. cervi* (Ács *et al*., 2016). Thus, the species seems to be present not only in Eastern Europe but also in various other locations worldwide. Infection with *D. cervi* has been associated with various manifestations of lung pathology, including interstitial pneumonia, bronchitis and bronchiolitis with an influx of eosinophils, lymphocytes, plasma cells and macrophages; massive hyperplasia of lymphoid follicles in bronchiolar tissue and hyperplasia of bronchial and bronchiolar epithelium (Pyziel *et al*., 2018*a*).

*D. cervi* differs from *D. eckerti* regarding the absence of cervical papillae, the presence of a single ring of 4 symmetrical submedian cephalic papillae, the length of the tail in females, the morphometry of the female reproductive organs and the dimensions of the gubernacula in males (Pyziel *et al*., 2017). Nevertheless, it should be kept in mind that in wild cervids, there is a higher risk of misidentification of *Dictyocaulus* when the identification is based solely on morphology (Divina *et al*., 2000). Therefore, it is recommended to complement the morphological examination with genetic analysis to enable accurate identification (Nadler and Pérez-Ponce de León, 2011).

Taxonomic differentiation of *Dictyocaulus* spp. can be facilitated by using the small subunit (*SSU*) and *ITS*2 ribosomal rDNA as genetic markers (Epe *et al*., 1997, Hӧglund *et al*., 1999; Pyziel, 2014), as well as more variable mitochondrial (*mt*) DNA markers (Hӧglund *et al*., 2006; Gasser *et al*., 2012). Our previous studies suggest that *SSU* of ribosomal rDNA and cytochrome B (*cyt*B) of *mt* DNA are well conserved, and they seem to be the most suitable markers for systematic and molecular epidemiological studies of *Dictyocaulus* spp. (Pyziel *et al*., 2020). The sequences of other *mt* markers are more variable within lungworm species, and are therefore more suitable for studying population genetics (Hu *et al*., 2002; Hӧglund *et al*., 2006; Gasser *et al*., 2012; Ács *et al*., 2016; Pyziel *et al*., 2018*a*).

Although knowledge about the species composition and genetic variability of *Dictyocaulus* spp. continues to increase, studies still report the emergence of new, enigmatic genotypes of lungworms in wild ruminants (Ács *et al*., 2016; Cafiso *et al*., 2023). It appears that the species composition and genetic variability within the genus *Dictyocaulus* continues to expand (Bangoura *et al*., 2021; Danks *et al*., 2022); for example, a lungworm with different *ITS*2 sequence data has been discovered in fallow deer in Sweden (Divina *et al*., 2002). The present study investigates the distribution, morphology and genetic diversity of *Dictyocaulus* spp. in red deer in Poland and in red deer, fallow deer and moose in Sweden.

## Materials and methods

### Specimen collection

The study was conducted on 167 individuals of red deer culled during 2017/2018 and 2018/2019 hunting seasons in Poland as part of a management strategy. Lungs, together with trachea, were collected from 65 animals from the Lower Silesian Wilderness (51° N 15° E, south-western Poland), 83 from the Bieszczady Mountains (49° N 22° E, south-eastern Poland), and 19 as control material from the Piska Forest (53° N 22° E, north-eastern Poland): an area where *D. cervi* was previously recorded as the only *Dictyocalus* species infecting red deer (Pyziel *et al*., 2017). Briefly, all red deer were dissected and their trachea, bronchi and bronchioles were cut open. Any adult *Dictyocaulus* sp. specimens were collected in laboratory tubes containing 70% ethanol and taken for testing.

### DNA extraction, amplification and sequencing

Depending on the intensity of infection, 1 to 5 male lungworms from each respiratory tract of the red deer examined were subjected to molecular analysis. Genomic DNA was extracted individually from 80 adult male lungworms using a Nucleospin tissue DNA extraction kit (Macherey-Nagel, Düren, Germany) according to the manufacturer's protocol.

In addition, DNA samples of lungworms were derived from 7 fallow deer, 4 red deer and 2 moose from Sweden collected for a previous study (Höglund *et al*., 2003). Regions of the small subunit ribosomal RNA (*SSU*), and mitochondrial cytochrome B (*cyt*B) of the worms were amplified by PCR using the following primers sets : NF50 (5′-TGA AAC TGC GAA CGG CTC AT-3′) + BNR1 (5'-ACC TAC AGA TAC CTT GTT ACG AC-3') for *SSU* (Pyziel *et al*., 2017); cytB_F (5'-TGA AAA RGT TAA GAT RRT TGG GAC-3') + cytB_R (5'-TTA GGA ATA GCA CGC AAA ATA GC-3') for *cyt*B (Pyziel *et al*., 2020). Genetic markers were selected based on previous studies (Pyziel *et al*., 2018*b*, 2020). Primers were designed using FastPCR software, version 5.4 (Primer Digital, Helsinki, Finland).

PCR was performed in a 2720 thermal cycler (Applied Biosystems, Foster City, California) in a 50 *μ*l volume. Each 50 *μ*l PCR reaction contained 20 *μ*l of Molecular Biology Reagent Water (Sigma-Aldrich, USA), 25 *μ*l Quant-Bio's AccuStart^TM^ II PCR ToughMix® ( × 2 concentration) (Quantabio, Beverly, USA), 1 *μ*l GelTrack Loading Dye (×50 concentration) (Quantabio, Beverly, USA), 1 *μ*l forward primer (20 mm), 1 *μ*l reverse primer (20 mm) and 2 *μ*l template DNA.

The conditions for PCR were as follows: 94°C for 2 min to denature DNA, with 35 cycles at 94°C for 40 s (SSU)/45 s (*cyt*B), 55°C for 1.5 min (SSU)/56°C for 60 s (*cyt*B), and 72°C for 2 min (SSU)/45 s (*cyt*B); with a final extension of 10 min at 72°C to ensure complete amplifications.

The PCR product was purified with the Nucleospin Gel and PCR Clean-up Kit (Macherey-Nagel, Germany), eluted with 30 *μ*l Molecular Biology Reagent Water (Sigma-Aldrich, USA) and sequenced in both directions by Macrogen Europe (Amsterdam, the Netherlands) using the primers (5 mm) used for amplification. The sequences were then assembled into contigs using CodonCode Aligner version 8.0 (CodonCode Corporation, Massachusetts, USA).

### Phylogenetic analysis

Phylogenetic analysis of the taxa of *Dictyocaulus* was performed by alignment with the partial nucleotide sequences of *SSU* (1,605 bp) and the amino acid sequences of the partial nucleotide sequences of *cyt*B (642 bp), and using the GTR + I (*cyt*B) and GTR + G (*SSU*) evolutionary model. A list of taxa included in the molecular analyses is listed in Table S1 (*SSU*), Table S2 (*cyt*B). Evolutionary models were selected based on JModelTest 2.1.10 (Guindon and Gascuel, 2003; Darriba *et al*., 2012) using the AIC criterion. The phylogenetic tree was constructed using Bayesian inference analysis (BI) with MrBayes version 3.2. (Huelsenbeck and Ronquist, 2001).

### Morphological characteristics

Molecular preselection led to the identification of a new lungworm genotype in 12 of the 64 *Dictyocaulus*-positive red deer: 4 individuals from the Lower Silesian Wilderness and 8 from the Bieszczady Mountains. These animals served as source for 54 nematodes (19 males and 35 females) which were examined morphologically. The worms were cleared in lactophenol and mounted on slides in glycerine jelly. Measurements and microphotographs were made using an Opta-Tech Lab40 light microscope (×40 –×1000 magnification) and the OptaView IS-PL Opta-Tech software package (Opta-Tech, Warsaw, Poland).

The following measurements were taken: body length, buccal capsule (BC), buccal capsule wall (BCW), head, cephalic vesicle and oesophagus of males and females; copulatory bursa, gubernaculum, spicules of the male reproductive system; vestibules, anterior sphincter and infundibulum, posterior sphincter and infundibulum, tail and phasmids of the female reproductive system. The location of the nerve ring, excretory pore and opening of the vulva were also examined.

Specimens destined for scanning electron microscopy (s.e.m.) were dehydrated in increasing concentrations of ethanol and stored in acetone (Eisenback, 1985). The dehydrated specimens were then dried at the critical-point with liquid CO_2_; their proximal endings were then cut and mounted on a s.e.m. mounting stub with double-coated adhesive tape, sputter-coated with gold, and examined with a LEO 1430VP (ZEISS, Jena, Germany) scanning electron microscope.

### Statistical analysis

The range, mean and standard deviation of the morphological measurements of *D. skrjabini* n. sp. were compared with those of *D. cervi* presented by Pyziel AM *et al*. (2017). The normality of each variable was first checked with the Shapiro–Wilk test.

The following morphological traits showed a normal distribution: length of the body, cephalic vesicle, BCW, anterior to nerve ring, anterior to excretory pore, copulatory bursa, gubernaculum, spicules, posterior to vulva opening, vestibules, anterior sphincter, anterior infundibulum, posterior sphincter, posterior infundibulum, posterior to anus (tail), posterior to phasmids; the width of BC, BCW, oesophagus max., body at vulva opening. These were compared using the Student's *T* test.

Other morphological traits, namely the length of the oesophagus and BC, and the width of the head and cephalic vesicle, were compared with the Mann–Whitney U-test. Probability values less than 0.05 (P < 0.05) were considered statistically significant.

## Results

### Prevalence and intensity of infection

*Dictyocaulus* spp. lungworms were diagnosed in 27 of 65 respiratory tracts of red deer from the Lower Silesian Wilderness (prevalence = 41.5%), in 26 of 83 from the Bieszczady Mountains (prevalence = 31.3%), and in 11 of 19 from the Piska Forest (prevalence = 57.9%). The mean intensity of infection was 13.7 ± 19.9 in the Lower Silesian Wilderness (range = 1–78), 11.1 ± 20.6 in the Bieszczady Mountains (range = 1–98), and 12.6 ± 10.5 from the Piska Forest (range = 1–39).

Both the red deer populations from the Lower Silesian Wilderness and from the Bieszczady Mountains were infected with *D. cervi* and a new species of large lungworm (*Dictyocaulus skrjabini* n. sp.); however, the red deer from the Piska Forest harboured *D. cervi* exclusively. None of the animals from the Lower Silesian Wilderness or the Bieszczady Mountains was found to have mixed infection with the 2 species: each *Dictyocaulus* spp. – positive red deer was infected with only one species. In both areas, *D. cervi* was overwhelmingly more prevalent in red deer than *D. skrjabini* n. sp.

*Dictyocaulus skrjabini* n. sp. was found in the respiratory tract of 4 individuals from the Lower Silesian Wilderness (prevalence = 6.1%), and in 8 from the Bieszczady Mountains (prevalence = 9.6%). The mean intensity of infection with *D. skrjabini* n. sp. was 7.5 ± 4.5 in the Lower Silesian Wilderness (range = 4–14), and 19.5 ± 32.8 in the Bieszczady Mountains (range = 1–98). The remaining *Dictyocaulus* belonged to *D. cervi* species.

### DNA sequences

The study revealed 186 new nucleotide sequences of the marker genes *SSU* and *mt cyt*B. Thirteen slightly different sequences derived from different hosts were submitted to GenBank [MN448405 – MN448408, MH756628, MH756629 (*SSU*); MN503296 – MN503299, MN503302 – MN503304 (*cyt*B)]. The *SSU* sequences varied in length from 1,639 base pairs (bp) to 1,652 bp, and *cyt*B from 693 bp to 744 bp.

In Sweden, all 7 isolates of fallow deer lungworms (GenBank: MN448406, MN503299, MN450300) and one isolate of lungworms derived from red deer (GenBank: MN503303) contained *D. skrjabini* n. sp. In addition, 3 isolates of lungworm from red deer (GenBank: MN503302, MN448405) and 2 from moose, i.e. from both studied individuals (GenBank: MN448407), were found to be *D. cervi*.

For the 1,605 bp *SSU* sequence, *D. skrjabini* n. sp. differed by 14 nucleotides from *D. cervi* and by 15 nucleotides from *D. eckerti* (0.9% sequence divergence) (Table 1).

**Table 1.** Pairwise comparison of small subunit rDNA sequence variability among 6 species (9 selected isolates) of *Dictyocaulus*

	Species	1	2	3	4	5	6	7	8	9
1	*D. filaria*		85 (5.30)	89 (5.54)	89 (5.54)	87 (5.42)	96 (5.98)	97 (6.04)	82 (5.11)	83 (5.17)
2	*D. skrjabini* n. sp.	85 (5.30)		17 (1.06)	17 (1.06)	17 (1.06)	30 (1.87)	31 (1.93)	14 (0.87)	15 (0.93)
3	*D. viviparus*	89 (5.54)	17 (1.06)		0	19 (1.18)	36 (2.42	37 (2.30)	11 (0.68)	12 (0.75)
4	*D. viviparus bisontis*	89 (5.54)	17 (1.06)	0		19 (1.18)	36 (2.42	37 (2.30)	11 (0.68)	12 (0.75)
5	*D. capreolus* 1	87 (5.42)	17 (1.06)	19 (1.18)	19 (1.18)		20 (1.25)	21 (1.31)	13 (0.81)	14 (0.87)
6	*D. capreolus* 2	96 (5.98)	30 (1.87)	36 (2.42)	36 (2.42)	20 (1.25)		1 (0.06)	30 (1.87)	31 (1.93)
7	*D. capreolus* 3	97 (6.04)	31 (1.93)	37 (2.30)	37 (2.30)	21 (1.31)	1 (0.06)		31 (1.93)	32 (1.99)
8	*D. cervi*	82 (5.11)	14 (0.87)	11 (0.68)	11 (0.68)	13 (0.81)	30 (1.87)	31 (1.93)		1 (0.06)
9	*D. eckerti*	83 (5.17)	15 (0.93)	12 (0.75)	12 (0.75)	14 (0.87)	31 (1.93)	32 (1.99)	1 (0.06)	

Number of variable sites in 1,605 base pair. Percentage of variable sites between 2 species/isolates is given in parentheses.

1: AJ920362, 2: MH756629, 3: AJ920361, 4: KC771250, 5: AY168859, 6:MG833326, 7: MG833324, 8: MT919232, 9: AY168864.

As for the 642 bp *cyt*B sequence, the nucleotide sequence variation within the species ranged from 2 to 6 nucleotides depending on the *D. skrjabini* n. sp. isolate (0.3–0.9% of sequence divergence). However, no variability was observed in the 214 amino acid sequence (Table 2). The *cyt*B gene sequence differed by 106–111 nucleotides between *D. skrjabini* n. sp. and *D. cervi* (16.5–17.3% nucleotide sequence divergence).

**Table 2.** Pairwise comparison of cytochrome b mitochondrial DNA nucleotide sequence and inferred amino acid sequence variability among 4 species (14 selected isolates) of *Dictyocaulus*

	Species	1	2	3	4	5	6	7	8	9	10	11	12	13	14
1	*D. cervi* 1		2 (0.31)	4 (0.62)	10 (1.56)	8 (1.25)	10 (1.56)	73 (11.37)	109 (16.98)	111 (17.29)	107 (16.66)	87 (13.55)	88 (13.71)	95 (14.80)	95 (14.80)
2	*D. cervi* 2	1 (0.47)		4 (0.62)	10 (1.56)	8 (1.25)	10 (1.56)	73 (11.37)	109 (16.98)	111 (17.29)	107 (16.66)	87 (13.55)	88 (13.71)	95 (14.80)	93 (14.26)
3	*D. cervi* 3	1 (0.47)	1 (0.47)		8 (1.25)	8 (1.25)	10 (1.56)	71 (11.06)	108 (16.82)	110 (17.13)	106 (16.51)	89 (13.86)	90 (14.02)	95 (14.80)	95 (14.80)
4	*D. cervi* 4	1 (0.47))	1 (0.47)	0		11 (1.71)	7 (1.09)	72 (11.21)	110 (17.13)	112 (17.44)	108 (16,82)	91 (14.17)	92 (14.33)	99 (15.42)	99 (15.42)
5	*D. cervi* 5	2 (0.93)	2 (0.93)	1 (0.47)	1 (0.47)		10 (1.56)	74 (11.53)	113 (17.60)	115 (17.91)	111 (17.29)	90 (14.02)	91 (14.17)	98 (15.26)	98 (15.26)
6	*D. cervi* 6	1 (0.47)	1 (0.47)	0	0	1 (0.47)		73 (11.37)	110 (17.13)	112 (17.44)	108 (16,82)	89 (13.86)	90 (14.02)	97 (15.11)	97 (15.11)
7	*D. eckerti*	13 (6.07)	13 (6.07)	12 (5.61)	12 (5.61)	13 (6.07)	12 (5.61)		121 (18.85)	122 (19.00)	120 (18.69)	103 (16.04)	104 (16.20)	103 (16.04)	103 (16.04)
8	*D. skrjabini* n. sp. 1	40 (18.69)	40 (18.69)	39 (18.22)	39 (18.22)	40 (18.69)	39 (18.22)	40 (18.69)		2 (0.31)	6 (0.93)	113 (17.60)	114 (17.76)	114 (17.76)	115 (17.91)
9	*D. skrjabini* n. sp. 2	40 (18.69)	40 (18.69)	39 (18.22)	39 (18.22)	40 (18.69)	39 (18.22)	40 (18.69)	0		4 (0.62)	115 (17.91)	116 (18.07)	116 (18.07)	117 (18.22)
10	*D. skrjabini* n. sp. 3	40 (18.69)	40 (18.69)	39 (18.22)	39 (18.22)	40 (18.69)	39 (18.22)	40 (18.69)	0	0		115 (17.91)	116 (18.07)	116 (18.07)	117 (18.22)
11	*D. viviparus* 1	24 (11.21)	24 (11.21)	23 (10.75)	23 (10.75)	23 (10.75)	23 (10.75)	26 (12.15)	42 (19.63)	42 (19.63)	42 (19.63)		1 (0.16)	13 (2.02)	13 (2.02)
12	*D. viviparus* 2	24 (11.21)	24 (11.21)	23 (10.75)	23 (10.75)	23 (10.75)	23 (10.75)	26 (12.15)	42 (19.63)	42 (19.63)	42 (19.63)	0		12 (1.87)	12 (1.87)
13	*D. viviparus bisontis* 1	27 (12.62)	27 (12.62)	26 (12.15)	26 (12.15)	26 (12.15)	26 (12.15)	27 (12.62)	44 (20.56)	44 (20.56)	44 (20.56)	4 (1.87)	4 (1.87)		2 (0.31)
14	*D. viviparus bisontis* 2	27 (12.62)	26 (12.15)	27 (12.62)	27 (12.62)	27 (12.62)	27 (12.62)	28 (13.08)	45 (21.03)	45 (21.03)	45 (21.03)	5 (2.34)	5 (2.34)	1 (0.47)	

Above diagonal = number of variable sites in 642 base pairs. Below diagonal = number of variable sites of 214 amino acids. Percentage of variable sites between 2 species/isolates is given in parentheses.

1: MN503304, 2: MN503296; 3: MN503302, 4: MT920216, 5: MT920217, 6: MT920218, 7: JX519459, 8: MN503303, 9: MN503297, 10: MN503299, 11: MN503298, 11: JX519460, 12: AP017683, 13: MN503300; 14: MN503301.

When the nucleotide sequences were translated into amino acid sequences, they differed by 39–40 amino acids (18.2–18.7% of amino acid sequence divergence). The nucleotide sequence divergence in the *cyt*B gene between *D. skrjabini* n. sp. and *D. eckerti* ranged from 120 to 122 nucleotides (i.e. 18.7–19% divergence); this corresponds to a difference of 40 amino acids between them (i.e. 18.7% divergence) (Table 2).

### Phylogenetic reconstruction

**Figure 1.**
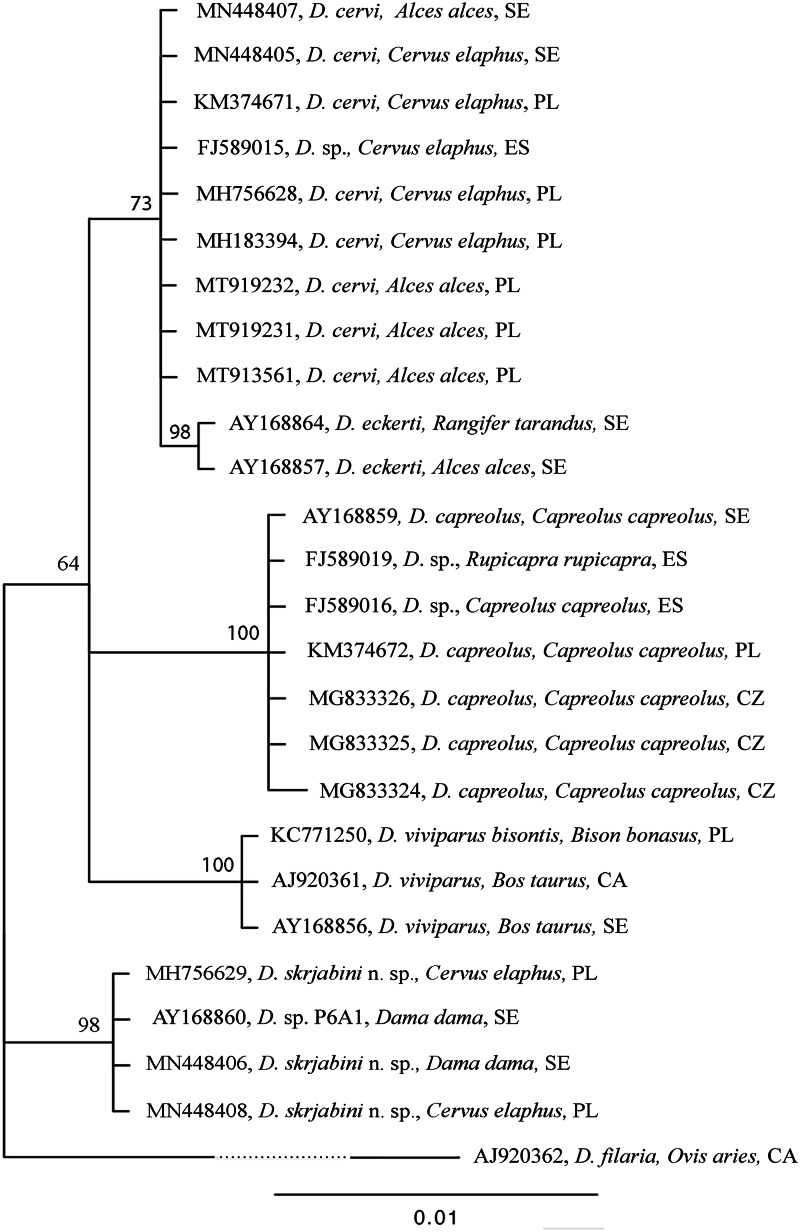
Phylogenetic tree of *Dictyocaulus* spp. based on *SSU* rDNA partial sequences, constructed with the use of Bayesian inference (BI) analysis using MrBayes version 3.2. The GTR + G model was chosen based on jModelTest version 2.1.4 using Akaike information criterion. The analysis was run for 1 000 000 generations, with 500 000 generations discarded as ‘burn-in’. GenBank accession numbers, hosts and country of origin are shown. Nodal support is indicated as Bayesian posterior probabilities. Sequence from *Dictyocaulus filaria* (AJ920362) was used as an outgroup.

Bayesian analysis (BI) of the *SSU* rDNA sequence data, with *Dictyocaulus filaria* as an outgroup, revealed 3 strongly supported clades (Fig. 1). One clade containing *D. filaria*, the second clade containing *D. skrjabini* n. sp. taxa from red deer and fallow deer, and the third clade containing 3 subclades: one subclade containing *D. viviparus* from cattle and European bison, the other subclade containing *D. capreolus* and *Dictyocaulus* sp. isolates from roe deer, the third subclade containing *D. cervi* from red deer and moose. In the third subclade, *D. eckerti* was a sister taxon of *D. cervi*.

**Figure 2.**
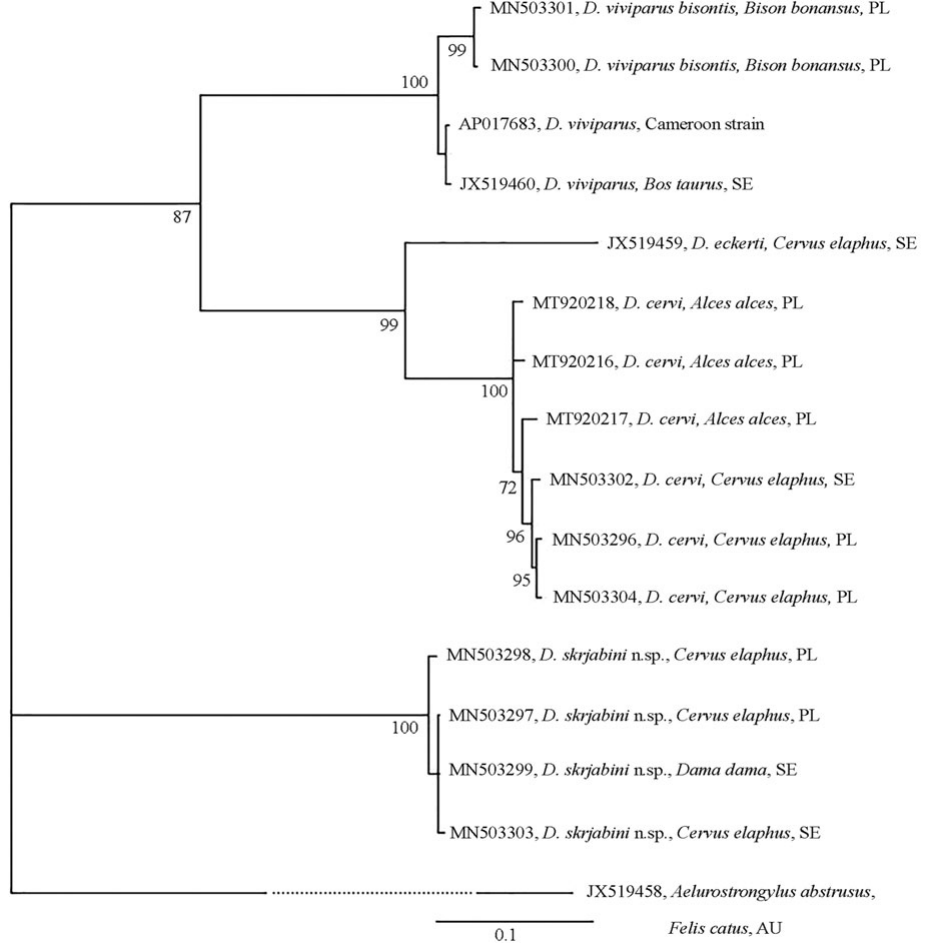
Phylogenetic tree of *Dictyocaulus* spp. based on *cyt*B partial sequences, constructed with the use of Bayesian inference (BI) analysis using MrBayes version 3.2. The GTR + I model was chosen based on jModelTest version 2.1.4 using Akaike information criterion. The analysis was run for 1 000 000 generations, with 500 000 generations discarded as ‘burn-in’. GenBank accession numbers, hosts and country of origin are shown. Nodal support is indicated as Bayesian posterior probabilities. Sequence from *Aelurostrongylus abstrusus* (JX519458) was used as an outgroup.

In the BI analysis of *cyt*B sequence data with *Aelurostrongylus abstrusus* as the outgroup, *D. skrjabini* n. sp. and *A. abstrusus* formed 2 independent clades (Fig. 2). The third cluster comprised 2 subclades: a subclade with *D. viviparus* from cattle and European bison, and a subclade with *D. eckerti* from red deer with *D. cervi* as a sister taxon. The *D. cervi* sequences were found to be heterogeneous.

### Description of *Dictyocaulus skrjabini* n. sp.

**Figure 3.**
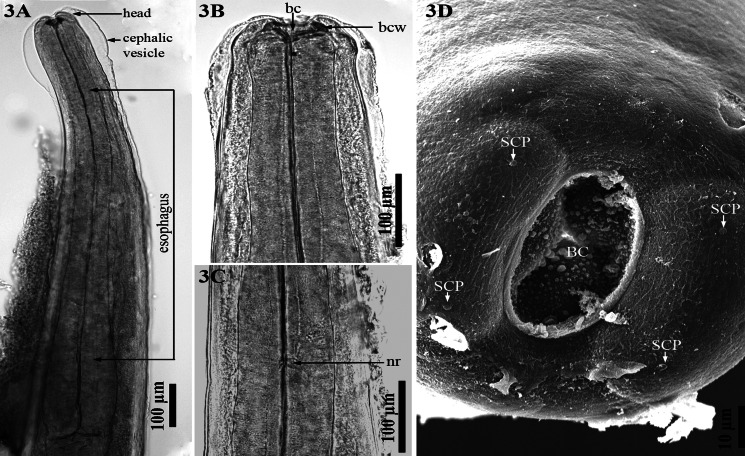
*Dictyocaulus skrjabini* n. sp. of red deer, anterior end. **(A)** Anterior end in optical section, showing head, cephalic vesicle, oesophagus, lateral view. **(B)** Anterior end in optical section, showing buccal capsule (bc), buccal capsule wall (bcw), cephalic vesicle, lateral view. **(C)** Anterior end below head region in optical section, showing nerve ring (nr), lateral view. **(D)** Cephalic region, scanning electron microscopy, showing BC and 4 submedian papillae (SCP).

*General morphology* (based on 54 specimens: 19 males and 35 females): BC oval, dorsoventrally flattened (Figs 3A, B, D). Oral opening elongate oval, dorsoventrally flattened. Thickness of BCW (range = 42–96 *μ*m) thick, according to Divina *et al*. (2000). Single ring of 4 symmetrical submedian cephalic papillae, lateral amphids absent (Fig. 3D). Cuticle with numerous longitudinal ridges. Cervical papillae absent, nerve ring (Fig. 3C) and excretory pore difficult to discern.

**Figure 4.**
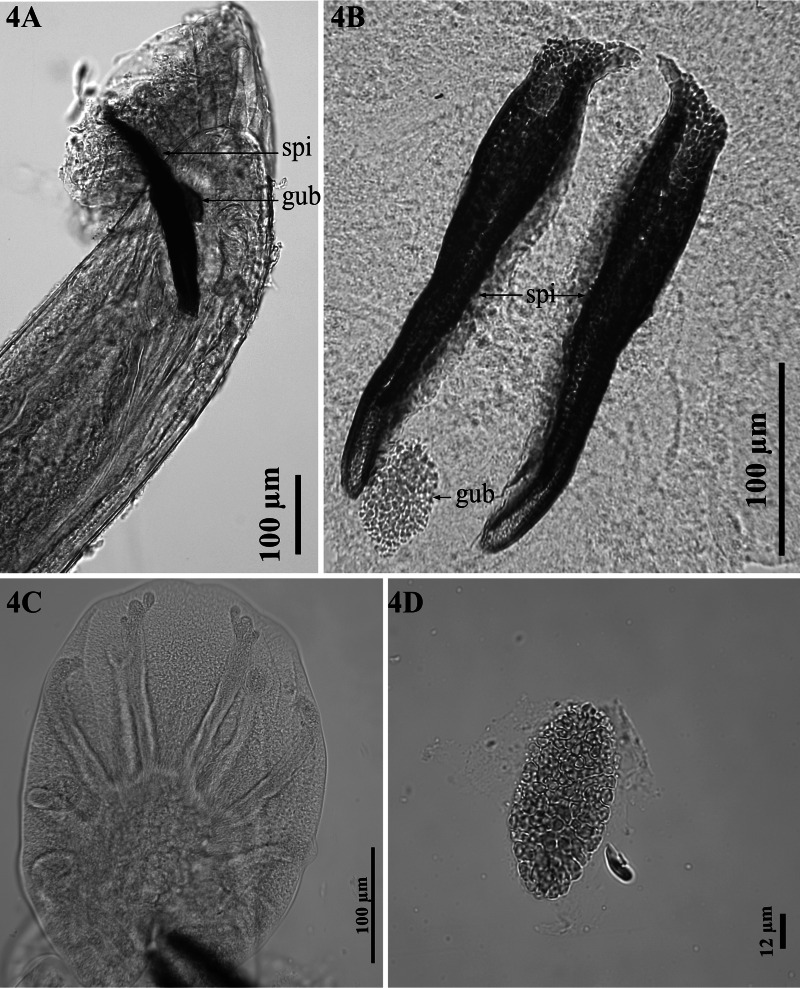
*Dictyocaulus skrjabini* n. sp. of red deer, male genital system, light microscopy. **(A)** Bursa, showing left spicula (spi) and gubernaculum (gub), lateral view. **(B)** Spicules (spi), gubernaculum (gub), dorsal view. **(C)** Bursa, abdominal view. **(D)** Gubernaculum, dorsal view.

*Male (holotype):* Body 8.2–46.6 mm long. Head 91–144 *μ*m wide. Cephalic vesicle present, 126–207 *μ*m long and 17–45 *μ*m wide. Oesophagus 901–1,449 *μ*m long, 117–297 *μ*m maximum width. BC 14–39 *μ*m wide, 40–53 *μ*m long. BCW 5–9 *μ*m wide and 52–96 *μ*m long. Anterior to nerve ring 348–491 *μ*m. Copulatory bursa 133–523 *μ*m long (Fig. 4A). Spicules 195–306 *μ*m long, porous texture, dark brown, small easily visible transparent membrane around the tip of each spicule (Fig. 4B). Gubernaculum present, 46–71 *μ*m long, 25–36 *μ*m width, porous texture, paler than spicules, irregularly oval in dorsoventral view, longitudinal, slightly curved at distal end, which is thinner than proximal end in lateral view (Figs 4A, B, D). Bursa bell shaped, lobes not separated, heart-shaped in dorsoventral view (Fig. 4C). Ventral rays parallel, with short common stem, anteroventral shorter than posteroventral, reaching about two-thirds of its length, not reaching bursal margin, posteroventral almost reaching bursal margin; anterolateral ray separate, short, not reaching bursal margin, with rounded distal tip; mediolateral and posterolateral rays completely fused, long, almost reaches bursal margin (Fig. 4C); externodorsal ray separate from, and shorter than, dorsal ray, with rounded distal tip; dorsal ray divided at base, each branch with 3 small divisions at distal tip, almost reaches bursal margin.

**Figure 5.**
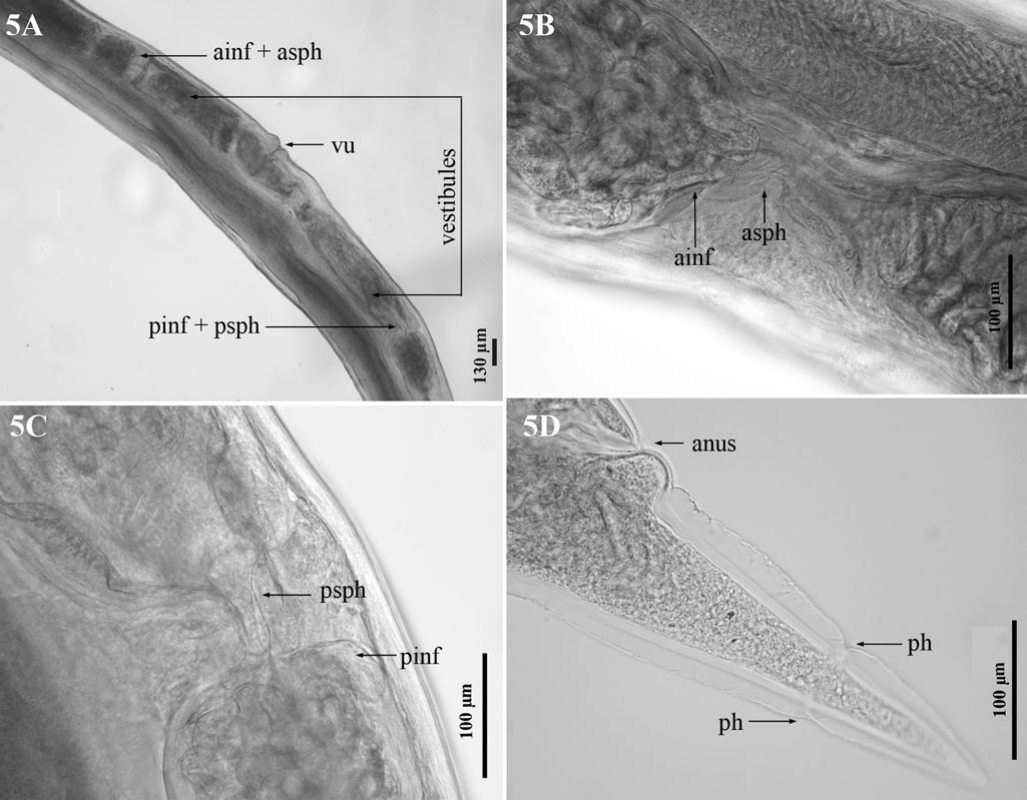
*Dictyocaulus skrjabini* n. sp. of red deer, female genital system, light microscopy. **(A)** Ovejectors in right lateral view, showing relationships for the vulva (vu), vestibules and combined anterior infundibulum and sphincter (ainf + asph) and posterior infundibulum and sphincter (pinf + psph). **(B)** Region of anterior infundibulum (ainf) and anterior sphincter (asph), right lateral view. **(C)** Region of posterior infundibulum (pinf) and posterior sphincter (psph), right lateral view. **(D)** Female tail, right lateral view, showing anus and phasmids (ph).

*Female (allotype):* Body 20.1–60.7 mm long. Head 96–183 *μ*m wide. Cephalic vesicle present, 162–219 *μ*m long and 18–35 *μ*m wide. Oesophagus 893–1,514 *μ*m long and 121–231 *μ*m maximum width. BC 16–49 *μ*m wide, 36–81 *μ*m long. BCW 4–10 *μ*m wide and 42–96 *μ*m long. Anterior to nerve ring 356–463 *μ*m. Vulva opening 18–25.2 mm from posterior end, vulval lips are slightly swollen (Fig. 5A). Body width at vulval opening 421–661 *μ*m. Reproductive apparatus didelphic, amphidelphic (Fig. 5A). Combined length of opposed vestibules 1,189–2,523 *μ*m. Length of anterior sphincter 58–167 *μ*m and length of anterior infundibulum 23–62 *μ*m (Fig. 5B). Length of posterior sphincter 48–114 *μ*m and length of posterior infundibulum 37–74 *μ*m (Fig. 5C). Tail 313–483 *μ*m long; phasmids 132–210 *μ*m from posterior end (Fig. 5D).

### Taxonomic summary

*Type host:* red deer, *Cervus elaphus* (Linnaeus, 1758) (Artiodactyla: Cervidae).

*Other known host:* fallow deer, *Dama dama* (Linnaeus, 1758) (Artiodactyla: Cervidae).

*Site of infection:* trachea, bronchi, bronchioles.

*Type locality:* Bieszczady Mountains, Poland (49° N 22° E).

*Distribution:* Lower Silesian Wilderness, Poland (51° N 15° E), Sweden (lack of detailed information).

*Deposited specimens:* Museum of Natural History, University of Wroclaw, Poland; holotype (male) (from Bieszczady Mountains, Poland) No. MNHW 1436a, and allotype (female) (from Lower Silesian Wilderness, Poland) No. MNHW 1436b.

*Prevalence of infection:* In 12 of 167 (7.2%).

*Deposited sequences:* GenBank MH756629 [*SSU* rDNA; *Cervus elaphus*; Bieszczady Mountains, Poland (49° N 22° E)], MN448408 [*SSU* rDNA; *Cervus elaphus*; Lower Silesian Wilderness, Poland (51° N 15° E)], MN472750 [*ITS*2 rDNA; *Cervus elaphus*; Bieszczady Mountains, Poland (49° N 22° E)], MN503298 [*mt*-*cyt*B; *Cervus elaphus*; Bieszczady Mountains, Poland (49° N 22° E)], MN503297 [*mt*-*cyt*B; *Cervus elaphus*; Lower Silesian Wilderness, Poland (51° N 15° E)], MN448406 (*SSU* rDNA; *Dama dama*; Sweden), MN503299 (*mt*-*cyt*B; *Dama dama*; Sweden), MN450300 (*ITS*2 rDNA; *Dama dama*; Sweden), MN503303 (*mt*-*cyt*B; *Cervus elaphus*; Sweden).

*Etymology:* The specific epithet derives from the name of professor Konstantin Ivanovich Skrjabin, a veterinary parasitologist whose study led to the description of *Dictyocaulus eckerti* from reindeer, *Rangifer tarandus*. Since then *D. eckerti* was maintained as a collective species infecting various cervid hosts. This arrangement was actual until *D. capreolus* and *D. cervi* were described and segregated from *D. eckerti*.

## Remarks

According to Divina *et al*. (2000), *Dictyocaulus* lungworms can be distinguished from each other morphologically mainly by BCW thickness and length. However, there are currently no available data on the BCW of *D. eckerti*, which makes any comparison with other *Dictyocaulus* species impossible (Pyziel *et al*., 2017). In the present study, the BCW of *D. skrjabini* n. sp. was statistically significantly longer and narrower than that of *D. cervi*. The mean length and width of the BCW of *D. skrjabini* was 63.4 ± 12.5 and 6.7 ± 1.1 in males, whereas it was 65 ± 10.3 and 7.3 ± 1.4 in females, respectively (Tables S3 and S5). The mean length and width of the BCW of *D. cervi* was 19.5 ± 6.4 and 7.8 ± 1.7 in males, whereas it was 23.9 ± 5.4 and 8.2 ± 1.5 in females, respectively (Pyziel *et al*., 2017). Moreover, according to Divina *et al*. (2000), the BCW was described as thick in *D. skrjabini* n. sp. and as medium in *D. cervi*. Although, a single ring of 4 symmetrical submedian cephalic papillae was observed in both *D. skrjabini* n. sp. and *D. cervi*, no lateral amphids were seen in *D. skrjabini* n. sp. compared to *D. cervi,* which bore 2 lateral amphids at its anterior extremity. In contrast, 2 rings of size-differentiated cephalic papillae were observed in *D. eckerti* consisting of 4 papillae in the external ring and 6 smaller papillae in the internal ring (Skrjabin *et al*., 1954). Furthermore, in contrast to *D. eckerti*, no cervical papillae were observed in either *D. skrjabini* n. sp. or *D. cervi*, (Skrjabin *et al*., 1954; Gibbons and Khalil, 1988). The nerve ring was visible in both *D. skrjabini* n. sp. and *D. cervi*, but not observed in *D. eckerti* (Gibbons and Khalil, 1988). Furthermore, no statistically significant differences in the length of the spicules were observed between *D. skrjabini* n. sp. and *D. cervi*, which is consistent with previous observations that the length of the spicules is not a reliable morphological trait for *Dictyocaulus* spp. (Gibbons and Khalil, 1988; Divina *et al*., 2000).

Females of *D. skrjabini* n. sp. were significantly shorter than females of *D. cervi*; however, no statistically significant difference in total body length was found between males of the 2 species (Table S3). Similarly, females of *D*. *skrjabini* n. sp. were wider at the vulval opening compared to those of *D. cervi* (Table S4). Regardless of sex, individuals of *D. skrjabini* n. sp. were characterized by a longer cephalic vesicle, BC, BCW, and a greater distance from the anterior extremity to the nerve ring compared to individuals of *D. cervi* (Table S3). The distance from the anterior extremity to the excretory pore was longer in the males of *D. skrjabini* n. sp., but shorter in the females of *D. skrjabini* n. sp., compared to individuals of *D. cervi* (Table S3). In both males and females of *D. skrjabini* n. sp. the head and oesophagus were wider than in *D. cervi* (Table S5). In contrast, cephalic vesicle, BC and BCW were narrower in *D. skrjabini* n. sp. compared to *D. cervi*, regardless of sex (Table S5).

The only statistically significant difference in the male reproductive system concerned the length of the gubernaculum, which was shorter in *D. skrjabini* n. sp. than in *D. cervi* (Table S6). In the female reproductive system, both vestibules and the anterior infundibulum were shorter in *D*. *skrjabini* n. sp. than in *D. cervi*; while the anterior sphincter and posterior sphincter were longer in *D. skrjabini* n. sp. (Table S4).

## Discussion

The genus *Dictyocaulus* was established by Railliet and Henry (1907) for large lung nematodes recovered from respiratory tract of artiodactylids. It was originally considered to have 4 species, namely *D. viviparus* (specific to cattle), *D. filaria* (specific to sheep and goat), *D. arnfieldi* (specific to donkeys and horses) and *D. noerneri* (specific to cervids). According to the first systematic revision of the genus (Skrjabin *et al*., 1954), *D. cameli* (specific to camels) was established and *D. noerneri* was replaced with *D. eckerti*, as *D. noerneri* was considered invalid. The second systematic revision of the genus *Dictyocaulus* (Gibbons and Khalil, 1988) resulted in description of *D. africanus* (specific to African artiodactylids). Since its erection, *D. eckerti* was maintained as a collective species infecting various cervid hosts including reindeer, red deer, elk, roe deer, moose, fallow deer, black sika deer, and hog deer (Skrjabin *et al*., 1954; Romano and Persiani, 1982; Gibbons and Khalil, 1988; Jansen and Borgsteede, 1990). This arrangement was actual until *D. capreolus* (Gibbons and Hӧglund, 2002) from roe deer and moose was described. Since the description of *D. capreolus*, 2 other species of large lungworms have been identified in cervids and distinguished from *D. eckerti*; namely *D. cervi* (Pyziel *et al*., 2017) and *D. skrjabini* n. sp. (the species described in this study). The genetic variability of red deer-derived *Dictyocaulus* spp. has been revealed in previous studies, suggesting that several distinct species may be concealed within *D. eckerti* (Carreno *et al*., 2009; Pyziel *et al*., 2015; Ács *et al*., 2016; Cafiso *et al*., 2023). In most cases, their nucleotide sequences were homologous to *D. cervi*, suggesting that the species might have a wide distribution in red deer populations in Spain, Sweden, Poland, Hungary and Italy (Carreno *et al*., 2009; Pyziel *et al*., 2015; Ács *et al*., 2016; Cafiso *et al*., 2023). Furthermore, *D. cervi* has also been detected in moose in Poland (Filip-Hutsch *et al*., 2020), as well as in rocky mountain elk in the USA (Bangoura *et al*., 2021). In addition, *D. cervi*, new genotypes of red deer lungworm have recently been detected in Italy (Cafiso *et al*., 2023).

In the present study, *D. cervi* was more prevalent than *D. skrjabini* n. sp., which accounted for only 18.7% of all positive *Dictyocaulus* samples from Poland. Molecular detection of *D. cervi* was also recorded in red deer and moose in Sweden, while *D. skrjabini* n. sp. was confirmed in all isolates of fallow deer and from one red deer examined. Interestingly, a new genotype of a lungworm previously found by Hӧglund *et al*. (2003) in fallow deer in Sweden showed 100% *SSU* rDNA homology with *D. skrjabini* n. sp.

The analysis of the molecular data, and their phylogenetic reconstruction revealed clear distinctions between *D. cervi*, *D. skrjabini* n. sp. and *D. eckerti*. *Dictyocaulus skrjabini* n. sp. formed independent clades in both phylogenetic trees, one based on *SSU* rDNA and the other on *mt cyt*B sequences. The results further highlight the high degree of conservation within the *SSU* rDNA and *mt cyt*B sequences of *Dictyocaulus* spp. and confirm their usefulness for systematic studies within the genus. Previous studies have indicated a high level of *Dictyocaulus* spp. genetic diversity within the genus at the *ITS*2 rDNA, and *cox*1, *cox*3 and *nad*5 of the *mt* DNA (Hӧglund *et al*., 2006; Pyziel *et al*., 2017; Pyziel *et al*., 2018*a*; Pyziel *et al*., 2020). In addition, the *cox*3 nucleotide sequences were found not to be suitable for lungworm species identification, as these sequences were identical for *D. viviparus* and *D. capreolus*: 2 different worm species (Pyziel *et al*., 2018*a*). According to Blouin (1998), different species of closely related nematodes can show 10–20% variation in *mt* gene sequences, as indicated for *D. skrjabini* n. sp. and *D. cervi*, and for *D. skrjabini* n. sp. and *D. eckerti* in the present study.

Additionally, morphological differences between *D. skrjabini* n. sp. and *D. cervi* included statistically significant discrepancies in various features.

Thus, a large lungworm found in red deer and fallow deer demonstrated clear molecular and morphological differences from previous specimens, suggesting the presence of a new species: *D. skrjabini* n. sp. The geographical distribution of the species requires further investigation. The exact localization of the new species in the lung is also of interest, both to better understand the drivers of speciation and to explain the pathogenicity of the parasite.

## Data Availability

The data that support the findings of this study are available from the corresponding author (AMP).

## Supplementary material

For supplementary material accompanying this paper visit https://doi.org/10.1017/S003118202300080X. click here to view supplementary material
