# Supplementary material for: Distribution of large lungworms (Nematoda: Dictyocaulidae) in free-roaming populations of red deer Cervus elaphus (L.) with the description of Dictyocaulus skrjabini n. sp
Source: Parasitology. 2023 Aug 24;150(10):956–66. doi: 10.1017/S003118202300080X (PMC10577652; doi:10.1017/S003118202300080X)
Supplement: Supplementary file 1 [file S003118202300080Xsup.zip › S003118202300080Xsup001.docx]

| **Species** | **Host** | **Country** | **GenBank** |
| --- | --- | --- | --- |
| *Dictyocaulus cervi* | *Cervus elaphus* | Sweden | MN448405 |
| *D. cervi* | *Cervus elaphus* | Poland | KM374671 |
| *D. cervi* | *Cervus elaphus* | Poland | MH756628 |
| *D. cervi* | *Cervus elaphus* | Poland | MH183394 |
| *D. cervi* | *Alces alces* | Sweden | MN448407 |
| *D. cervi* | *Alces alces* | Poland | MT919232 |
| *D. cervi* | *Alces alces* | Poland | MT919231 |
| *D. cervi* | *Alces alces*  *Cervus elaphus* | Poland  Spain | MT913561  FJ589015 |
| *D.* sp. |  |  |  |
| *D. eckerti* | *Rangifer tarandus* | Sweden | AY168864 |
| *D. eckerti* | *Alces alces* | Sweden | AY168857 |
| *D. capreolus* | *Capreolus capreolus* | Sweden | AY168859 |
| *D. cepreolus* | *Capreolus capreolus* | Poland | KM374672 |
| *D. cepreolus* | *Capreolus capreolus* | Czech Rep. | MG833326 |
| *D. cepreolus* | *Capreolus capreolus* | Czech Rep. | MG833325 |
| *D. cepreolus* | *Capreolus capreolus* | Czech Rep. | MG833324 |
| *D.* sp. | *Rupicapra rupicapra* | Spain | FJ589019 |
| *D.* sp. | *Capreolus capreolus* | Spain | FJ589016 |
| *D. viviparus bisontis* | *Bison bonasus* | Poland | KC771250 |
| *D. viviparus* | *Bos taurus* | Canada | AJ920361 |
| *D. viviparus* | *Bos taurus* | Sweden | AY168856 |
| *D. skrjabini* n. sp. | *Cervus elaphus* | Poland | MH756629 |
| *D*. sp*.* P6A1 | *Dama dama* | Sweden | AY168860 |
| *D. skrjabini* n. sp. | *Dama dama* | Sweden | MN448406 |
| *D. skrjabini* n. sp. | *Cervus elaphus* | Poland | MN448408 |
| *D. filaria* | *Ovis aries* | Canada | AJ920362 |

**Table S1.** List of taxa included in the molecular analysis using *SSU* rDNA sequence data.
