# Supplementary material for: Distribution of large lungworms (Nematoda: Dictyocaulidae) in free-roaming populations of red deer Cervus elaphus (L.) with the description of Dictyocaulus skrjabini n. sp
Source: Parasitology. 2023 Aug 24;150(10):956–66. doi: 10.1017/S003118202300080X (PMC10577652; doi:10.1017/S003118202300080X)
Supplement: Supplementary file 1 [file S003118202300080Xsup.zip › S003118202300080Xsup002.docx]

| **Species** | **Host** | **Country** | **GenBank** |
| --- | --- | --- | --- |
| *D. viviparus bisontis* | *Bison bonasus* | Poland | MN503301 |
| *D. viviparus bisontis* | *Bison bonasus* | Poland | MN503300 |
| *D. viviparus* | No data available | Cameroon | AP017683 |
| *D. viviparus* | *Bos taurus* | Sweden | JX519460 |
| *D. eckerti* | *Cervus elaphus* | Sweden | JX519459 |
| *D. cervi* | *Alces alces* | Poland | MT920218 |
| *D. cervi* | *Alces alces* | Poland | MT920216 |
| *D. cervi* | *Alces alces*  *Cervus elaphus* | Poland  Sweden | MT920217  MN503302 |
| *D. cervi* |  |  |  |
| *D. cervi* | *Cervus elaphus* | Poland | MN503296 |
| *D. cervi* | *Cervus elpahus* | Poland | MN503304 |
| *D. skrjabini* n. sp. | *Cervus elaphus* | Poland | MN503298 |
| *D. skrjabini* n. sp. | *Cervus elaphus* | Poland | MN503297 |
| *D. skrjabini* n. sp. | *Dama dama* | Sweden | MN503299 |
| *D. skrjabini* n. sp. | *Cervus elaphus* | Sweden | MN503303 |
| *Aelurostrongylus abstrusus* | *Felis catus* | Australia | JX519458 |

**Table S2.** List of taxa included in the molecular analysis using mitochondrial *cyt*B sequence. data.
