# Supplementary material for: Distribution of large lungworms (Nematoda: Dictyocaulidae) in free-roaming populations of red deer Cervus elaphus (L.) with the description of Dictyocaulus skrjabini n. sp
Source: Parasitology. 2023 Aug 24;150(10):956–66. doi: 10.1017/S003118202300080X (PMC10577652; doi:10.1017/S003118202300080X)
Supplement: Supplementary file 1 [file S003118202300080Xsup.zip › S003118202300080Xsup003.docx]

**Table S3.** The length characterization of the morphological features of *Dictyocaulus* *skrjabini* n. sp. compared with *D. cervi* (Pyziel *et al.* 2017); all dimensions are given in μm except body length, given in mm; T test: Student's t-test, M-W test: Mann–Whitney U test.

| FEATURE | | *Dictyocaulus skrjabini* n. sp. | | *D. cervi* | | *D. skrjabini* *vs*. *D. cervi* | |
| --- | --- | --- | --- | --- | --- | --- | --- |
|  |  | male | famale | male | female | male | female |
| Body | Range  Sample size (*n*)  Mean ± standard deviation | 8.2-46.6  19  35.6±11.3 | 20.1-60.7  35  46.3±10.3 | 25.6-56.7  19  40.2±7.9 | 30.3-76.8  34  52.7±9.8 | *p*=0.078  (T test) | *p*=0.005*  (T test) |
| Cephalic vesicle | Range  Sample size (*n*)  Mean ± standard deviation | 126-207  9  157.9±27.1 | 162-219  6  188.2±22.6 | 110.5-232,6  23  148.1±30.7 | 137.1-207.7  21  166.1±20.1 | *p*=0.232  (M-W test) | *p*=0.015*  (T test) |
| Buccal capsule | Range  Sample size (*n*)  Mean ± standard deviation | 40-53  12  46.3±4.2 | 36-81  29  51.5±9.6 | 11.6-29.2  16  19.9±5.6 | 14.2-39.2  32  21.9±6.2 | *p*<0.001*  (T test) | *p*<0.001*  (M-W test) |
| Buccal capsule wall | Range  Sample size (*n*)  Mean ± standard deviation | 52-96  12  63.4±12.5 | 42-96  25  65±10.3 | 10.7-33.5  22  19.5±6.4 | 14.5-30.7  24  23.9±5.4 | *p*<0.001*  (T test) | *p*<0.001*  (T test) |
| Oesophagus | Range  Sample size (*n*)  Mean ± standard deviation | 901-1,449  24  1,209.9±138.5 | 893-1,514  39  1,234.5±159.5 | 824.5-1,309.7  23  1,064.3±116.3 | 856.5-1,452.5  41  1,149.4±145.5 | *p*<0.001*  (T test) | *p*=0.008*  (M-W test) |
| Anterior to nerve ring | Range  Sample size (*n*)  Mean ± standard deviation | 348-491  18  414.3±37.4 | 356-463  28  419.4±30.6 | 297.9-423.3  20  364.3±35.7 | 262.5-466.3  36  388.1±40.2 | *p*<0.001*  (M-W test) | *p*<0.001*  (T test) |
| Anterior to excretory pore | Range  Sample size (*n*)  Mean ± standard deviation | 478-596  3  537.7±59 | 368-524  6  462±57.3 | 326.5-544.1  21  443.8±50.5 | 421.3-581.9  28  499.7±41.9 | *p*=0.026*  (M-W test) | *p*=0.035*  (T test) |

*statistically significant difference
