# Supplementary material for: Distribution of large lungworms (Nematoda: Dictyocaulidae) in free-roaming populations of red deer Cervus elaphus (L.) with the description of Dictyocaulus skrjabini n. sp
Source: Parasitology. 2023 Aug 24;150(10):956–66. doi: 10.1017/S003118202300080X (PMC10577652; doi:10.1017/S003118202300080X)
Supplement: Supplementary file 1 [file S003118202300080Xsup.zip › S003118202300080Xsup004.docx]

**Table S4.** Dimensional characterization of the morphological features of the female reproductive system of *Dictyocaulus* *skrjabini* n. sp. compared with *D. cervi* (Pyziel *et al.* 2017); all dimensions are given in μm except posterior to vulva opening, given in mm; T test: Student's t-test.

| FEATURE | | *D. skrjabini* n. sp. | | | *D. cervi* | *D. skrjabini* vs. *D. cervi* |
| --- | --- | --- | --- | --- | --- | --- |
| Posterior to vulva opening | Range  Sample size (*n*)  Mean ± standard deviation | | 18–25.2  ­15  22.9±1.9 | | 13.3–33  36  23.2±4.5 | *p=*0.378  (T test) |
| Body width at vulva opening | Range  Sample size (*n*)  Mean ± standard deviation | | 421–661  21  520.7±68.7 | | 304.8–549.8  36  453.3±69.1 | *p<*0.001*  (T test) |
| Length of vestibules | Range  Sample size (*n*)  Mean ± standard deviation | | 1,189–2,523  19  1,935.8±358.7 | | 1,115.3–2,810.8  40  2,194.5±410.9 | *p=*0.011*  (T test) |
| Length of anterior sphincter | Range  Sample size (*n*)  Mean ± standard deviation | | 58–167  20  92.5±28.1 | | 41–94.2  47  67.3±13.7 | *p<*0.001*  (T test) |
| Length of anterior infundibulum | Range  Sample size (*n*)  Mean ± standard deviation | | 23–62  10  49.1±11.7 | | 38.8–78.1  47  61.2±11 | *p=*0.001*  (T test) |
| Length of posterior sphincter | Range  Sample size (*n*)  Mean ± standard deviation | | 48-114  28  77.3±16.5 | | 47.3-87.2  45  66.8±10.7 | *p=*0.002*  (T test) |
| Length of posterior infundibulum | Range  Sample size (*n*)  Mean ± standard deviation | | 37-74  12  51.2±10.2 | | 32.1-81.8  43  55.1±10.7 | *p=*0.128  (T test) |
| Posterior to anus  (length of tail) | Range  Sample size (*n*)  Mean ± standard deviation | | | 313–483  16  358.2±43.9 | 283.8–475.5  30  356±43.2 | *p=*0.417  (T test) |
| Posterior to phasmids | Range  Sample size (*n*)  Mean ± standard deviation | | | 132–210  24  160.7±20.3 | 116.2–226.7  54  156.2±22.2 | *p=*0.202  (T test) |
|  |  | | |  |  |  |

*statistically significant differences
