# Supplementary material for: Distribution of large lungworms (Nematoda: Dictyocaulidae) in free-roaming populations of red deer Cervus elaphus (L.) with the description of Dictyocaulus skrjabini n. sp
Source: Parasitology. 2023 Aug 24;150(10):956–66. doi: 10.1017/S003118202300080X (PMC10577652; doi:10.1017/S003118202300080X)
Supplement: Supplementary file 1 [file S003118202300080Xsup.zip › S003118202300080Xsup005.docx]

**Table S5.** The width characterization of the morphological features of red deer-derived *Dictyocaulus* *skrjabini* n. sp. compared with *D. cervi* (Pyziel *et al.* 2017); T test: Student's t-test, M-W test: Mann–Whitney U test.

| FEATURE | | *Dictyocaulus skrjabini* n. sp. | | *Dictyocaulus cervi* | | *D. skrjabini* vs. *D. cervi* | |
| --- | --- | --- | --- | --- | --- | --- | --- |
|  |  | male | famale | male | female | male | female |
| Head | Range  Sample size (*n*)  Mean ± standard deviation | 91-144  11  118.1±16.1 | 96-183  15  123.9±24.4 | 77.9-113.6  24  93.9±9.5 | 69.1-128.2  41  103.1±13.4 | *p<*0.001*  (T test) | *p<*0.001***  (M-W test) |
| Cephalic vesicle | Range  Sample size (*n*)  Mean ± standard deviation | 17-45  9  29.3±10.6 | 18-35  5  24.8±8.4 | 111.7-168.2  20  136±14.4 | 120-191  15  148.6±21.4 | *p<*0.001*  (T test) | *p=*0.001*  (M-W test) |
| Buccal capsule | Range  Sample size (*n*)  Mean ± standard deviation | 14-39  11  31.3±7.8 | 16-49  26  33.5±7.4 | 25.1-54.3  23  39.2±7.4 | 31.4-55  32  44.1±7.3 | *p=*0.003*  (T test) | *p<*0.001*  (T test) |
| Buccal capsule wall | Range  Sample size (*n*)  Mean ± standard deviation | 5-9  18  6.7±1.1 | 4-10  49  7.3±1.4 | 5-12.2  43  7.8±1.7 | 5.3-12.5  73  8.2±1.5 | *p=*0.021*  (M-W test) | *p<*0.001*  (T test) |
| Oesophagus max. | Range  Sample size (*n*)  Mean ± standard deviation | 117-297  24  178.8±40.2 | 121-231  37  187.5±29.4 | 105.4-172.3  23  138.3±16.8 | 106.5-220.2  36  157.3±25.1 | *p<*0.001*  (M-W test) | *p<*0.001*  (T test) |

*statistically significant difference
