# Supplementary material for: Distribution of large lungworms (Nematoda: Dictyocaulidae) in free-roaming populations of red deer Cervus elaphus (L.) with the description of Dictyocaulus skrjabini n. sp
Source: Parasitology. 2023 Aug 24;150(10):956–66. doi: 10.1017/S003118202300080X (PMC10577652; doi:10.1017/S003118202300080X)
Supplement: Supplementary file 1 [file S003118202300080Xsup.zip › S003118202300080Xsup006.docx]

**Table S6.** Dimensional characterization of the morphological features of the male reproductive system of *Dictyocaulus* *skrjabini* n. sp. compared with *D. cervi* (Pyziel *et al.* 2017); all dimensions are given in μm; T test: Student's t-test.

| FEATURE |  | *Dictyocaulus skrjabini* n. sp. | *D. cervi* | *D. skrjabini* vs. *D. cervi* |
| --- | --- | --- | --- | --- |
| Length of copulatory bursa | Range  Sample size (*n*)  Mean ± standard deviation | 133-523  13  270±94.9 | 171.8-282.3  16  229.5±28.2 | *p*=0.058  (T test) |
| Length of gubernaculum | Range  Sample size (*n*)  Mean ± standard deviation | 46-71  15  55.5±6.9 | 43-73  18  62.8±8.6 | *p*=0.011*  (T test) |
| Width of gubernaculum | Range  Sample size (*n*)  Mean ± standard deviation | 25-36  8  30.1±3.3 | 27.4-38.9  7  32.8±3.7 | *p*=0.079  (T test) |
| Length of spicules | Range  Sample size (*n*)  Mean ± standard deviation | 195-306  32  264.8±28.5 | 208.9-302.9  42  261.1±26.1 | *p*=0.283  (T test) |

*statistically significant differences
